# Topical Anti-inflammatory Activity of New Hybrid Molecules of Terpenes and Synthetic Drugs

**DOI:** 10.3390/molecules200611219

**Published:** 2015-06-18

**Authors:** Cristina Theoduloz, Carla Delporte, Gabriela Valenzuela-Barra, Ximena Silva, Solange Cádiz, Fernanda Bustamante, Mariano Walter Pertino, Guillermo Schmeda-Hirschmann

**Affiliations:** 1 Laboratorio de Cultivo Celular, Facultad de Ciencias de la Salud, Universidad de Talca, Casilla 747, Talca 3460000, Chile; 2 Laboratorio de Productos Naturales, Facultad de Ciencias Químicas y Farmacéuticas, Universidad de Chile, Santos Dumont 964, Independencia, Santiago 8380000, Chile; E-Mails: cdelpor@uchile.cl (C.D.); gabadriela@gmail.com (G.V.-B.); 3 Instituto de Salud Pública de Chile, Marathon 1000, Santiago 7750000, Chile; E-Mail: xsilva@ispch.cl; 4 Laboratorio de Química de Productos Naturales, Instituto de Química de Recursos Naturales, Universidad de Talca, Casilla 747, Talca 3460000, Chile; E-Mails: solange.cadiz@gmail.com (S.C.); nanda_danan@hotmail.com (F.B.); mwalter@utalca.cl (M.W.P.); schmeda@utalca.cl (G.S.-H.)

**Keywords:** terpenes, ibuprofen, naproxen, anti-inflammatory activity, basal cytotoxicity

## Abstract

The aim of the study was to assess changes in the activity of anti-inflammatory terpenes from Chilean medicinal plants after the formation of derivatives incorporating synthetic anti-inflammatory agents. Ten new hybrid molecules were synthesized combining terpenes (ferruginol (**1**), imbricatolic acid (**2**) and oleanolic acid (**3**)) with ibuprofen (**4**) or naproxen (**5**). The topical anti-inflammatory activity of the compounds was assessed in mice by the arachidonic acid (AA) and 12-*O*-tetradecanoyl phorbol 13-acetate (TPA) induced ear edema assays. Basal cytotoxicity was determined towards human lung fibroblasts, gastric epithelial cells and hepatocytes. At 1.4 µmol/mouse, a strong anti-inflammatory effect in the TPA assay was observed for oleanoyl ibuprofenate **12** (79.9%) and oleanoyl ibuprofenate methyl ester **15** (80.0%). In the AA assay, the best activity was observed for **12** at 3.2 µmol/mouse, with 56.8% reduction of inflammation, in the same range as nimesulide (48.9%). All the terpenyl-synthetic anti-inflammatory hybrids showed better effects in the TPA assay, with best activity for **6**, **12** and **15**. The cytotoxicity of the compounds **8** and **10** with a free COOH, was higher than that of **2**. The derivatives from **3** were less toxic than the triterpene. Several of the new compounds presented better anti-inflammatory effect and lower cytotoxicity than the parent terpenes.

## 1. Introduction

Traditional medicine in South America has a vast knowledge in the use of plants to maintain and restore health. In Chile, the Mapuche Amerindians used different plant parts or plant products to relieve inflammation or conditions associated with inflammation [1,2]. The resin of *Araucaria araucana* (Mol.) Koch (Araucariaceae), used in topical application for skin inflammation and bruises, contains imbricatolic acid and other labdane diterpenes [1]. The aerial parts of *Fabiana imbricata* Ruiz et Pav. (Solanaceae) were applied as a poultice for broken bones and contusions and contain the triterpene oleanolic acid [2]. Diterpenes and triterpenes are natural products with potential as anti-inflammatory agents. Labdane diterpenes derivatives showed anti-inflammatory activity in the ear edema assay in mice as well as in macrophages [3]. Labdanolic acid methyl ester exhibited anti-inflammatory effects both in animal and cell culture models [4]. The diterpene ferruginol, occurring in the aerial parts of *Prumnopitys andina* (Poepp. et Endl.) shows anti-inflammatory activity [5]. It presents strong inhibition of lipid peroxidation in erythrocytes [6] as well as on superoxide anion generated in phorbol myristate acetate-stimulated rat neutrophils [7]. The triterpene oleanolic acid has been shown to display several relevant biological activities, including the modulation of the immune-inflammatory response [8], reduction of differentiation markers in adipocytes and suppression of inflammation associated with obesity [9]. The anti-inflammatory and analgesic effect of semisynthetic oleanolic acid derivatives was recently reported [10]. Pentacyclic triterpene acids from *Ugni molinae* leaves presented topical anti-inflammatory effect in mice [11].

Little has been done on the synthesis and biological evaluation of hybrid molecules combining a naturally occurring terpene with anti-inflammatory effect and a synthetic anti-inflammatory drug. This approach, combining different chemical moieties, has been explored by our research group with terpenyl quinones of sesquiterpenes [12] and diterpenes [13]. The selected terpenes ferruginol (**1**), imbricatolic acid (**2**) and oleanolic acid (**3**) present a OH function that can be used to prepare derivatives with the commercial anti-inflammatory drugs ibuprofen and naproxen, leading to new hybrid compounds containing a terpene and a synthetic anti-inflammatory moiety.

Many diseases are associated with a considerable inflammatory response, indicative of a host defence mechanism. Inflammation, in this pathological context, has received considerable attention to design therapeutic strategies to minimize disease-associated morbidity and mortality. Acute inflammatory reactions are characterized by changes in vascular permeability and local hemodynamics resulting in edema and cellular influx [14]. Inflammatory models of several types allow hypothesis testing, evaluation of test compounds, and provide a better understanding of the inflammatory process. Arachidonic acid (AA) or 12-*O*-tetradecanoylphorbol 13-acetate (TPA), are widely used agents that induce cutaneous inflammation in experimental animals [11,14,15,16].

*In vitro* assays using mammalian cell cultures provide valuable information about the toxicity of compounds in a cost-effective way, avoiding excessive use of laboratory animals. The determination of the basal cytotoxicity of the compounds is a first step for further toxicity studies. Human cell cultures can be used as an approach to estimate acute toxicity [17] and are relevant to assess the effect of compounds as skin irritants [18].

The aim of the study was to disclose possible changes in the topical anti-inflammatory effect and cytotoxicity of terpenes obtained from some Chilean crude drugs (used as anti-inflammatory agents) when combined with synthetic anti-inflammatory compounds (ibuprofen and naproxen). The topical anti-inflammatory effect of studied compounds was evaluated in mice and the basal cytotoxicity of the compounds was assessed towards the following human cell lines: lung fibroblasts (MRC-5), gastric epithelial AGS cells and hepatocytes HepG2.

## 2. Results and Discussion

**Figure 1:**
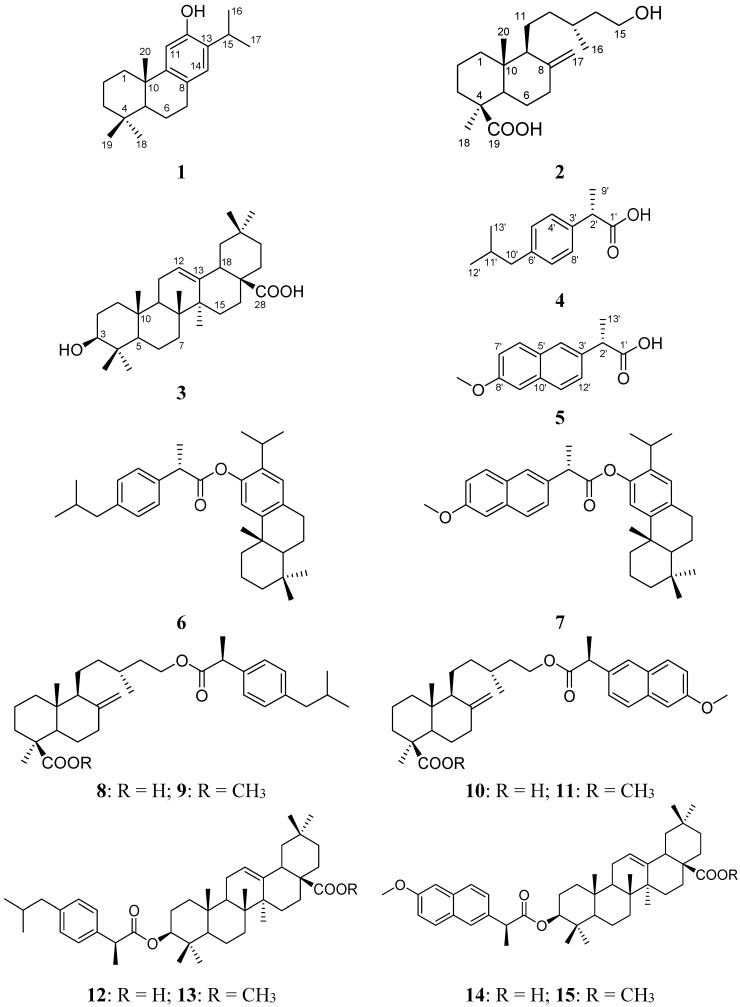
Structure of the starting terpenes **1**–**3**, synthetic anti-inflammatory drugs **4**, **5** and the new compounds **6**–**15**.

Starting from the terpenes ferruginol (**1**), imbricatolic acid (**2**) and oleanolic acid (**3**), and the commercial drugs ibuprofen (**4**) and naproxen (**5**), ten new terpenyl ibuprofenate and naproxenate derivatives were synthesized. The structures of the starting and new compounds are summarized in Figure 1. The percent *w*/*w* yields of the new synthetic compound were as follows: **6**: 67%; **7**: 71%; **8**: 64%; **9**: 88%; **10**: 56%; **11**: 89%; **12**: 62%; **13**: 92%; **14**: 61%; **15**: 85%. The spectroscopic and spectrometric data of the compounds were in agreement with the proposed structures.

The comparative topical anti-inflammatory effect of the compounds **1**–**15** was assessed in the model of ear edema induction in mice, using TPA and AA. This model is widely used for topical anti-inflammatory studies of synthetic and natural products and gives information on their probable action mechanism [19]. TPA induced inflammation develops more slowly than AA induced inflammation.

The topical administration of TPA provokes an acute edema with leukocyte infiltration, activating protein kinase C (PKC), which is Ca^2+^ and phospholipid dependent. PKC plays an important role in the signal transduction of a great variety of substances associated with cellular and proliferation functions. Active PKC acts at different levels, activating the nuclear factor kappa B (NF-κB). This transcription factor promotes the expression of several pro-inflammatory agents, such as cyclooxygenase 2 (COX-2) and inducible nitric oxide synthase (iNOS), inflammatory cytokines such as interleukins IL-1, IL-2, IL-6, IL-8 and the tumor necrosis factor TNF-α. Its excessive production leads to chronic pro-inflammatory diseases, leading to prostanoid synthesis and increased production of free radicals [16]. By contrast, the inflammatory response to AA is faster and is produced by an increased activity of myeloperoxidase and elastase, due to neutrophil arrival after applying this inflammatory agent [16]. Lipoxygenase inhibitors show higher potency than cyclooxygenase inhibitors on AA-induced edema, while cyclooxygenase inhibitors are more potent on TPA-induced edema [11,15,20].

The selection of the labdane diterpene imbricatolic acid, the triterpene oleanolic acid and the diterpene ferruginol was based on the traditional use of the plant drugs to treat inflammation and previous information on anti-inflammatory effect of the terpenes occurring in the plants.

The naturally occurring compounds **1**–**3** were assessed as anti-inflammatory agents by the mice ear edema model induced by AA and TPA. As ferruginol showed a significant effect in both models of inflammation (21.0 ± 2.1% edema reduction in the AA and 20.4 ± 3.1% edema reduction in the TPA model, respectively) and oleanolic acid presented a strong reduction of the ear edema in the TPA model (70.2 ± 8.3% edema reduction), we decided to prepare derivatives from the naturally occurring terpenes including in the new chemical entities a synthetic anti-inflammatory agent. The question to be answered was how far can the inclusion of a synthetic anti-inflammatory drug modify the activity of the natural products in the same experimental models of inflammation. Ibuprofen and naproxen were selected because they present a free carboxylic acid functions allowing the synthesis of ester derivatives with the selected terpenes. All the studied compounds were assessed as topical anti-inflammatory agents at a single dose, equimolar to the reference drugs (Table 1). Results are presented as percent decrease in the inflammatory response (edema) compared to controls treated only with the inflammatory agents. At 1.4 µmol/mouse, a strong anti-inflammatory effect in the TPA assay was observed for **12** (79.9%) and **15** (80.0%). The value for the reference compound (indomethacin) was 92.9%. In the AA assay, best activity was observed for **12** at 3.2 µmol/mouse, with 56.8% reduction of inflammation, in the same range as the reference compound nimesulide (48.9%). All the new compounds showed a better effect in the TPA assay, with best activity for compounds **12**, **14** and **15**.

**Table 1:** Topical anti-inflammatory effect of compounds **1**–**15** in the mice ear edema model induced by arachidonic acid (TA_AA_) and 12-*O*-tetradecanoylphorbol 13-acetate (TA_TPA_). Results are expressed as percent reduction of the ear edema.

Compounds	% TA_AA_ ± SEM	% TA_TPA_ ± SEM
*Terpenes*		
Ferruginol (**1**)	21.0 ± 2.1 *^,a^	20.4 ± 3.1 *^,a^
Imbricatolic acid (**2**)	5.0 ± 3.0	10.1 ± 4.0 *^a^*
Oleanolic acid (**3**)	2.7 ± 7.5	70.2 ± 8.3 *^,a^
*Synthetic anti-inflammatory compounds*		
Ibuprofen (**4**)	7.2 ± 5.5	56.2 ± 3.4 *^,a^
Naproxen (**5**)	1.8 ± 7.5 *^a^*	29.9 ± 6.2 *^,a^
*Esters*		
Ferruginyl ibuprofenate (**6**)	14.9 ± 7.1 *^,a^	47.9 ± 4.6 *^,a^
Ferruginyl naproxenate (**7**)	26.6 ± 4.1 *^,a^	26.8 ± 3.8 *^,a^
Imbricatol-15-yl ibuprofenate (**8**)	10.0 ± 6.0 *^a^*	40.3 ± 2.4 *^,a^
Imbricatol-15-yl ibuprofenate methyl ester (**9**)	14.9 ± 7.4 *	41.2 ± 6.2 *^,a^
Imbricatol-15-yl naproxenate (**10**)	16.3 ± 3.8 *^,a^	37.7 ± 3.3 *^,a^
Imbricatol-15-yl naproxenate methyl ester (**11**)	20.0 ± 9.4 *^,a^	42.7 ± 8.3 *^,a^
Oleanoyl ibuprofenate (**12**)	56.8 ± 7.4 *	79.9 ± 10.6 *
Oleanoyl ibuprofenate methyl ester (**13**)	19.5 ± 9.2 *^,a^	47.4 ± 2.6 *^,a^
Oleanoyl naproxenate (**14**)	9.0 ± 11.3 *^a^*	55.5 ± 5.1 *^,a^
Oleanoyl naproxenate methyl ester (**15**)	2.7 ± 8.1 *^a^*	80.0 ± 11.0 *
*Reference compounds*		
Nimesulide	↑48.9 ± 5.5 *	n.d. *^b^*
Indomethacin	n.d. *^b^*	↑92.9 ± 13.2 *

* p ≤ 0.05 compared with control group; *^a^* *p* ≤ 0.05 compared with reference drug; *n* = 8; ↑ Maximal effect; SEM standard error of the mean values. *^b^* n.d.: not determined. Mice were treated with compounds (**1**–**15**) or reference drugs at the equimolar dose of 3.2 µmol/mouse for the AA and 1.4 µmol/mouse for the TPA assays, respectively.

The activity of the ferruginol derivatives **6** and **7** in the TPA model was comparable with that of ibuprofen and naproxen, respectively. In the AA model, the effect was similar to that of the diterpene **1** alone. Therefore, the new compounds present activity on the AA induced inflammation, while ibuprofen and naproxen were inactive. When observing the anti-inflammatory effect of the new compounds, all derivatives were more effective than diterpene **2**, reducing inflammation by 10.0%–20.0% in the AA-induced and 37.7%–42.7% in the TPA-induced inflammation, respectively. The anti-inflammatory effect of the derivatives **10** and **11** with naproxen was higher than that of naproxen and much higher than that of **2** as a single molecule. Oleanolic acid (**3**) was active in the TPA assay but inactive in the AA model. However, the derivative **12** presented a significant effect in the AA assay.

Naproxen and ibuprofen attenuated the AA induced edema response, but did not affect the influx of neutrophils associated to an increase of myeloperoxidase (MPO). This explains the low anti-inflammatory response obtained for AA. Ibuprofen attenuated the TPA induced anti-inflammatory response with better activity than naproxen [14]. The reference drug indomethacin used in topical assays, is a potent potent inhibitor of cyclooxygenase isoenzymes (COX-1 and COX-2). Nimesulide is a weak inhibitor of cyclooxygenases [21]. Indomethacin is more potent in anti-inflammatory tests due to its ability to inhibit COXs [22], acting in the early stages of inflammation. Nimesulide is effective in the AA model and shows little, if any, activity against TPA-elicited inflammation. This could be explained, at least in part, if we consider nimesulide as a free radical scavenger [23].

The relevance of the carboxyl group for a strong anti-inflammatory effect, particularly against TPA, has been reported [24]. It is worth noting that **3** and both anti-inflammatory drugs **4** and **5** have the acid group, and all showed a significant anti-inflammatory effect against TPA. Therefore, we could assume that the acid group plays an important role in the topical anti-inflammatory activity. The synthesis of two cacalol esters of ibuprofen and naproxen has been described. However, when evaluated in the TPA-induced ear edema in mice, the compounds were inactive at the doses of 0.1 to 1 mg/ear [25]. The synthesis and analgesic effect of ibuprofen heterocyclic amides was reported [26]. The authors described analgesic effect of the amides in the acetic acid-induced writhing model after oral administration. Ibuprofen at 40 mg/kg reduced stretches by 77.6% and the most active new compounds by 96.5% and 89.7%, respectively. However, the activity investigated is different from that of our work. Ibuprofen glucopyranosides were synthesized and assessed in different models of inflammation, pain and gastric lesions [27]. In the xylene-induced ear edema in mice, the effect of ibuprofen and the derivatives was similar, with 45.9% reduction by ibuprofen and 52.7% for the most active ibuprofen propyl glucopyranoside ester. The authors concluded that the esterification of the free carboxylic acid of ibuprofen did not affect the anti-inflammatory and analgesic activity of the compound but suppressed gastric ulceration. Novel thiazolo[3,2-*b*]-1,2,4-triazole-6(5*H*)-one naproxen derivatives were synthesized and evaluated for anti-inflammatory and analgesic effect in mice [28]. In the carrageenan-induced paw edema, used to determine anti-inflammatory effect, oral administration of the compounds at 50 mg/kg showed for some of the new compounds an effect comparable to that of naproxen. None of the new compounds were more active than the parent compound in this assay. Our work showed that some of the new terpenyl hybrids of ibuprofen and naproxen were more active than the parent compounds. However, the experimental models used by other authors are different.

Human cell cultures as biological models are an efficient and complementary alternative to the use of laboratory animals, at least in a first step [17]. *In vitro* cytotoxicity methods reduce the number of laboratory animals, allow good data reproducibility and constitute a valuable tool to predict potential toxicity of the studied products prior to human testing [18]. The cytotoxicity of the compounds, expressed as IC_50_ values (µM), was determined on human lung fibroblasts (MRC-5), gastric epithelial cells (AGS) and hepatocytes HepG2. Cell viability was determined by means of the MTT reduction assay (Table 2). While the synthetic anti-inflammatory drugs ibuprofen and naproxen did not show toxicity towards the selected cell lines (IC_50_ > 1000 µM), the diterpenes **1** and **2** showed strong and moderate cytotoxic effect, respectively. The most cytotoxic compound was ferruginol **1** (IC_50_ values range: 29–39 µM). The new compounds **6** and **7** containing **1** as the terpene moiety showed a remarkable decrease in the cytotoxicity against the three cell lines, compared with the diterpene, with IC_50_ values ranging from 874 and >1000 µM. The derivatives **6** and **7**, with lower cell toxicity showed better effect as anti-inflammatory agents than ferruginol.

**Table 2:** Cytotoxicity of compounds **1**–**15** towards confluent cultures of three human cell lines: lung fibroblasts (MRC-5), gastric epithelial cells (AGS) and hepatocytes Hep G2.

Compounds	(IC_50_ ± SD, µM) *^a^*		
	MRC-5	AGS	HepG2
*Terpenes*			
Ferruginol (**1**)	31 ± 2	29 ± 1	39 ± 3
Imbricatolic acid (**2**)	134 ± 7	148 ± 9	111 ± 7
Oleanolic acid (**3**)	186 ± 9	234 ± 16	170 ± 5
*Synthetic anti-inflammatory compounds*			
Ibuprofen (**4**)	>1000	>1000	>1000
Naproxen (**5**)	>1000	>1000	>1000
*Esters*			
Ferruginyl ibuprofenate (**6**)	>1000	>1000	874 ± 34
Ferruginyl naproxenate (**7**)	945 ± 56	874 ± 49	>1000
Imbricatol-15-yl ibuprofenate (**8**)	34 ± 2	23 ± 1	79 ± 8
Imbricatol-15-yl ibuprofenate methyl ester (**9**)	706 ± 35	623 ± 39	746 ± 38
Imbricatol-15-yl naproxenate (**10**)	37 ± 2	29 ± 2	69 ± 4
Imbricatol-15-yl naproxenate methyl ester (**11**)	907 ± 54	601 ± 25	>1000
Oleanoyl ibuprofenate (**12**)	616 ± 32	526 ± 19	>1000
Oleanoyl ibuprofenate methyl ester (**13**)	402 ± 20	454 ± 21	742 ± 48
Oleanoyl naproxenate (**14**)	554 ± 22	486 ± 26	>1000
Oleanoyl naproxenate methyl ester (**15**)	816 ± 42	593 ± 31	>1000
Etoposide *^b^*	3.9 ± 0.1	0.36 ± 0.02	2.4 ± 0.1

*^a^* Results are expressed as mean values ± SD. Cells were treated during 24 h with the compounds. Cell viability was determined by means of the MTT reduction assay; *^b^* reference compound.

The diterpene **2** presents cytotoxicity with IC_50_ values ranging from 111 to 148 µM. The derivatives **8** and **10** (IC_50_ values 23–79 µM and 29–69 µM, respectively) were more cytotoxic than the starting natural product and the synthetic anti-inflammatory agents alone. No relevant differences in cytotoxicity were found between the compounds **8** and **10**, differing in the synthetic moiety of the molecule. The cytotoxicity of both compounds, however, is strongly reduced after methylation of the free COOH group to afford compounds **9** and **11**, with IC_50_ values in the range of 601 to >1000 µM. These findings show that the cytotoxicity of the new hybrids can be higher or lower than that of the starting terpenes, depending on structural features of the diterpene moiety. The difference between compounds **8** and **9** as well as **10** and **11** is the presence of a free carboxylic acid in the diterpene moiety of **8** and **10**. This functional group is protected as a methyl ester in compounds **9** and **11**. This fact indicates the relevance of the carboxylic acid in the biological effect. When cytotoxicity and anti-inflammatory effects are taken together, the best hybrids of this group were the compounds **9** and **11**, with lower cell toxicity and higher anti-inflammatory effect. The derivatives of oleanolic acid **3** were less toxic than the parent compound. Some of them presented higher anti-inflammatory activity than the synthetic products (compounds **12** and **15** in the TPA and compound **12** in the AA model). Little differences were found in cell toxicity of compounds **12** and **14**, with a free COOH function, compared with the methyl ester **13** and **15**, except against HepG2 cells where compounds **12**–**15** could be regarded as not toxic.

The results show that the anti-inflammatory effect of the naturally occurring terpenes **1**–**3** can be modulated when preparing hybrid molecules with a synthetic anti-inflammatory agent. The new products present different properties than the single molecule constituents, suggesting potential of further studies on structure-activity relationships of compounds combining natural products and bioactive synthetic moieties into new chemical entities. The low cytotoxicity of the active anti-inflammatory hybrids suggests a potential application in the treatment of inflammatory responses including allergic skin disorders and atopic dermatitis. Several of the esters presented remarkable increase in anti-inflammatory effect and lower cell toxicity than the parent compounds, supporting this approach in the search for new bioactive molecules.

## 3. Experimental Section

### 3.1. Equipment and General Procedures

Optical rotations were obtained for solutions in CHCl_3_ (concentrations expressed in g/100 mL) on a DIP 370 polarimeter (Jasco, Easton, MD, USA). IR spectra were recorded on a Nexus FT-IR instrument (Nicolet, Quebec, Canada). ^1^H-NMR spectra were recorded at 400 MHz and ^13^C-NMR data were obtained at 100 MHz on an Avance spectrometer (Bruker, Billerica, MA, USA). Chemical shifts are given in δ (ppm) with residual chloroform as the internal standard.

Mass spectra were measured in a LC/MSD-TOF (Agilent Technologies, Santa Clara, CA, USA) by the electrospray technique. Positive or negative ion mode was used according to the samples. As internal reference in the ESI (+) mode, purine (*m*/*z* 121.0509) and HP-0921 (*m*/*z* 922.0098) were used. In the negative mode: ESI (−), purine (*m*/*z* 112.9856) and HP-0921 (*m*/*z* 1033.9881) were used as internal references. Silica gel 60 (63–200 µm particle size, Merck, Kenilworth, NJ, USA) was used for column chromatography, pre-coated silica gel plates (Merck, Kieselgel 60 F254, 0.25 mm) were used for thin layer chromatography (TLC). TLC spots were visualized by spraying the chromatograms with *p*-anisaldehyde/ethanol/acetic acid/H_2_SO_4_ (2:170:20:10 *v*/*v*) and heating at 110 °C for 3 min. *N*,*N*-dicyclohexylcarbodiimide (DCC) and dimethylaminopyridine (DMAP) were from Sigma-Aldrich (St. Louis, MO, USA).

### 3.2. Preparation of Derivatives

#### 3.2.1. Starting Compounds **1**–**5**

The terpenes used for the synthesis were isolated from natural sources as reported in previous work. Ferruginol (**1**) was obtained from the bark of *Prumnopitys andina* (Poepp. et Endl.) de Laub (Podocarpaceae) according to [6,29]. Imbricatolic acid (**2**) was isolated from the resin of *Araucaria araucana* (Mol.) Koch (Araucariaceae) as described in [1]. Oleanolic acid (**3**) was isolated from the aerial parts of *Fabiana imbricata* Ruiz et Pav. (Solanaceae) and the procedure is described in [30]. The physical constants and spectroscopic data of compounds **1**–**3** are in full agreement with literature data. Detailed protocols for the isolation and purification are described in [1,6,29,30]. The compounds used for the synthesis were of purity higher than 95% as determined by NMR analysis and co-chromatography with pure standards obtained from the same plants. Racemic ibuprofen (**4**) and (*S*)-naproxen (**5**) with purities >95% were a gift from Laboratorio Chile (Santiago de Chile, Chile).

#### 3.2.2. Synthesis of Compounds **6**–**15**

*Ferruginyl ibuprofenate* (**6**): Ferruginol (177 mg, 0.62 mmol), DCC (191 mg, 0.93 mmol), a catalytic amount of DMAP and ibuprofen (127 mg, 0.62 mmol) were stirred at room temperature in dry CH_2_Cl_2_ (10 mL) for 2 h. The reaction was stopped by addition of water. The aqueous phase was extracted with EtOAc (3 × 20 mL), and dried over anhydrous Na_2_SO_4_, followed by evaporation of the solvent under reduced pressure. The residue was purified by silica gel column chromatography eluting with hexane/EtOAc (9:1), yielding **6** (195 mg, 67%). Pale yellow resin; Rf 0.65 (PE**/**EtOAc 95:5); ${\left[\alpha \right]}_{D}^{20}$ +37.1 (*c* 2.12, CHCl_3_); IR ν_max_ (film) 2959, 2925 (C–H, asymmetrical stretching, CH_3_ and CH_2_), 2861 (C–H symmetrical stretching, CH_3_ and CH_2_), 1756 (C=O, ester), 1163, 1141 (C–O, ester) cm^−1^; ^1^H-NMR (CDCl_3_): δ 6.88 (1H, s, H-14), 6.79 (1H, s, H-11), 3.22 (1H, m, H-15), 2.88, 2.83 (each 1H, m, H-7), 2.15 (1H, d, *J* = 11.6 Hz, H-1β), 1.18 (3H, s, H-20), 0.95 (3H, s, H-18), 0.94 (6H, d, *J* = 6.8 Hz, H-16 and H-17), 0.93 (3H, s, H-19), Ibuprofen moiety: δ 7.34 (2H, d, *J* = 8.0 Hz, H-4′ and H-8′), 7.16 (2H, d, *J* = 8.0 Hz, H-5′ and H-7′), 3.96 (1H, q, *J* = 7.1 Hz, H-2′), 2.50 (2H, d, *J* = 6.8 Hz, H-10′), 1.65 (3H, d, *J* = 7.2 Hz, H-9′), 0.98 (6H, d, *J* = 8.0 Hz, H-12′ and H-13′); ^13^C-NMR (CDCl_3_): δ 38.46 (C-1), 18.05 (C-2), 41.61 (C-3), 34.89 (C-4), 49.98 (C-5), 19.16 (C-6), 30.22 (C-7), 132.76 (C-8), 148.50 (C-9), 37.56 (C-10), 117.70 (C-11), 146.08 (C-12), 136.69 (C-13), 126.56 (C-14), 26.42 (C-15), 24.67 (C-16), 24.71 (C-17), 33.23 (C-18), 21.55 (C-19), 25.23 (C-20), Ibuprofen moiety: 173.33 (C-1′), 45.00 (C-2′), 137.36 (C-3′), 129.32 (C-4′), 127.38 (C-5′), 140.70 (C-6′), 127.38 (C-7′), 129.32 (C-8′), 18.99 (C-9′), 45.31 (C-10′), 29.91 (C-11′), 22.30 (C-12′), 22.30 (C-13′); HR-MS (EI) *m*/*z* 475.3556 [M + H]^+^ (calcd. for C_33_H_47_O_2_, 475.3576).

*Ferruginyl naproxenate* (**7**): Compound **7** was synthesized as described for **6** from ferruginol and naproxen to afford, after purification by silica gel column chromatography eluting with hexane/EtOAc (9:1), 251 mg (71%) of **7**. Pale yellow resin; Rf 0.62 (PE/DCM 1:1); ${\left[\alpha \right]}_{D}^{20}$ +56.4 (*c* 0.33, CHCl_3_); IR ν_max_ (film) 2953 (C–H, asymmetrical stretching, CH_3_ and CH_2_), 2859 (C–H symmetrical stretching, CH_3_ and CH_2_), 1750 (C=O, ester), 1266, 1138 (C–O, ester) cm^−1^; ^1^H-NMR (CDCl_3_): δ 6.88 (1H, s, H-14), 6.80 (1H, s, H-11), 2.55 (1H, m, H-15), 2.88, 2.82 (each 1H, m, H-7), 2.13 (1H, d, *J* = 11.6 Hz, H-1β), 1.17 (3H, s, H-20), 0.95 (3H, s, H-18), 0.94 (6H, d, *J* = 6.8 Hz, H-16 and H-17), 0.93 (3H, s, H-19), Naproxen moiety: δ 7.82 (1H, d, *J* = 2.4 Hz, H-4′), 7.77 (1H, d, *J* = 8.8 Hz, H-6′), 7.75 (1H, d, *J* = 8.8 Hz, H-11′), 7.55 (1H, dd, *J* = 8.8; 1.6 Hz, H-12′), 7.19 (1H, dd, *J* = 8.8; 2.4 Hz, H-7′), 7.17 (1H, br s, H-9′), 4.14 (1H, q, *J* = 6.8 Hz, H-2′), 3.96 (3H, s, OMe), 1.74 (3H, d, *J* = 6.8 Hz, H-13′); ^13^C-NMR (CDCl_3_): δ 37.96 (C-1), 19.42 (C-2), 42.04 (C-3), 33.69 (C-4), 50.47 (C-5), 19.60 (C-6), 30.38 (C-7), 133.31 (C-8), 149.01 (C-9), 39.10 (C-10), 118.15 (C-11), 146.53 (C-12), 137.12 (C-13), 126.73 (C-14), 26.97 (C-15), 23.01 (C-16), 23.34 (C-17), 33.82 (C-18), 22.00 (C-19), 25.14 (C-20), Naproxen moiety: 173.79 (C-1′), 46.10 (C-2′), 135.72 (C-3′), 126.77 (C-4′), 129.40 (C-5′), 129.70 (C-6′), 119.44 (C-7′), 158.06 (C-8′), 105.96 (C-9′), 134.24 (C-10′), 127.62 (C-11′), 127.04 (C-12′), 18.74 (C-13′), 55.72 (OMe′); HR-MS (EI) *m*/*z* 499.3196 [M + H]^+^ (calcd. for C_34_H_43_O_3_, 499.3212).

*Imbricatol-15-yl ibuprofenate* (**8**): Compound **8** was synthesized as described for **6** from imbricatolic acid and ibuprofen to afford, after purification by silica gel column chromatography eluting with hexane/EtOAc (8:2), 260 mg (64%) of **8**. Pale yellow resinous oil; Rf 0.64 (PE**/**EtOAc 7:3); ${\left[\alpha \right]}_{D}^{20}$ +19.7 (*c* 0.32, CHCl_3_); IR ν_max_ (film) 2956, 2928 (C–H, asymmetrical stretching, CH_3_ and CH_2_), 2848 (C–H, symmetrical stretching, CH_3_ and CH_2_), 1735 (C=O, ester), 1692 (C=O, carboxylic acid), 1166 (C–O, ester) cm^−1^; ^1^H-NMR (CDCl_3_): δ 4.85, 4.48 (each, 1H, br s, H-17), 4.11 (2H, m, H-15), 1.27 (3H, s, H-18), 0.93 (3H, d, *J* = 6.4 Hz, H-16), 0.62 (3H, s, H-20), Ibuprofen moiety: δ 7.22 (2H, d, *J* = 8.0 Hz, H-4′ and H-8′), 7.11 (2H, d, *J* = 8.0 Hz, H-5′ and H-7′), 3.70 (1H, q, *J* = 7.2 Hz, H-2′), 2.47 (2H, d, *J* = 7.2 Hz, H-10′), 1.51 (3H, d, *J* = 7.2 Hz, H-9′), 0.92 (6H, d, *J* = 6.8 Hz, H-12′ and H-13′); ^13^C-NMR (CDCl_3_): δ 39.53 (C-1), 20.07 (C-2), 38.35 (C-3), 45.62 (C-4), 56.78 (C-5), 26.45 (C-6), 39.17 (C-7), 148.57 (C-8), 56.97 (C-9), 40.96 (C-10), 21.47 (C-11), 36.45 (C-12), 30.62 (C-13), 35.61 (C-14), 63.66 (C-15), 20.01 (C-16), 106.85 (C-17), 29.47 (C-18), 184.81 (C-19), 13.18 (C-20), Ibuprofen moiety: 175.29 (C-1′), 44.63 (C-2′), 138.30 (C-3′), 129.69 (C-4′), 127.57 (C-5′), 140.84 (C-6′), 127.57 (C-7′), 129.69 (C-8′), 18.89 (C-9′), 45.47 (C-10′), 30.78 (C-11′), 22.83 (C-12′), 22.83 (C-13′); HR-MS (EI) *m*/*z* 509.3626 [M − H]^+^ (calcd. for C_33_H_49_O_4_, 509.3631).

*Imbricatol-15-yl ibuprofenate methyl ester* (**9**): **8** (100 mg, 0.20 mmol), was methylated with a solution of CH_2_N_2_ in diethyl ether. The solvent was evaporated under reduced pressure and the residue was purified by silica gel column chromatography eluting with hexane/EtOAc (9:1) yielding 90 mg (88%) of **9**. Pale yellow resinous oil; Rf 0.63 (PE:EtOAc 9:1); ${\left[\alpha \right]}_{D}^{20}$ +23.7 (*c* 0.30, CHCl_3_); IR ν_max_ (film) 2953 (C–H, asymmetrical stretching, CH_3_ and CH_2_), 2867 (C–H, symmetrical stretching, CH_3_ and CH_2_), 1729 (C=O, ester), 1166 (C–O, ester) cm^−1^; ^1^H-NMR (CDCl_3_): δ 4.74, 4.36 (each, 1H, br s, H-17), 4.00 (2H, m, H-15), 3.54 (3H, s, OMe), 1.11 (3H, s, H-18), 0.76 (3H, d, *J* = 6.4 Hz, H-16), 0.42 (3H, s, H-20), Ibuprofen moiety: δ 7.12 (2H, d, *J* = 8.0 Hz, H-4′ and H-8′), 7.01 (2H, d, *J* = 8.0 Hz, H-5′ and H-7′), 3.60 (1H, q, *J* = 7.2 Hz, H-2′), 2.37 (2H, d, *J* = 7.2 Hz, H-10′), 1.40 (3H, d, *J* = 7.2 Hz, H-9′), 0.82 (6H, d, *J* = 6.8 Hz, H-12′ and H-13′); ^13^C-NMR (CDCl_3_): δ 39.57 (C-1), 20.06 (C-2), 38.69 (C-3), 45.60 (C-4), 56.76 (C-5), 26.67 (C-6), 39.19 (C-7), 148.70 (C-8), 56.96 (C-9), 40.75 (C-10), 21.46 (C-11), 36.44 (C-12), 30.61 (C-13), 35.59 (C-14), 63.65 (C-15), 20.06 (C-16), 106.72 (C-17), 29.26 (C-18), 178.25 (C-19), 12.97 (C-20), 51.57 (OMe), Ibuprofen moiety: 175.25 (C-1′), 44.71 (C-2′), 138.26 (C-3′), 129.77 (C-4′), 127.55 (C-5′), 140.85 (C-6′), 127.55 (C-7′), 129.77 (C-8′), 18.88 (C-9′), 45.45 (C-10′), 30.78 (C-11′), 22.80 (C-12′), 22.80 (C-13′); HR-MS (EI) *m*/*z* 525.3920 [M + H]^+^ (calcd. for C_34_H_53_O_4_, 525.3944).

*Imbricatol-15-yl naproxenate* (**10**): Compound **10** was synthesized as described for **6** from imbricatolic acid and naproxen to afford, after purification by silica gel column chromatography eluting with hexane/EtOAc (8:2), 170 mg (56%) of **10**. Pale yellow resinous oil; Rf 0.49 (PE**/**EtOAc 7:3); ${\left[\alpha \right]}_{D}^{20}$ +22.2 (*c* 0.66, CHCl_3_); IR ν_max_ (film) 3000–3400 (O–H, from carboxylic acid), 2959, 2934 (C–H, asymmetrical stretching, CH_3_ and CH_2_), 2876 (C–H, symmetrical stretching, CH_3_ and CH_2_), 1732 (C=O, ester), 1692 (C=O, carboxylic acid), 1266, 1037 (C–O, ester) cm^−1^; ^1^H-NMR (CDCl_3_): δ 4.82, 4.43 (each, 1H, br s, H-17), 4.13 (2H, br t, *J* = 6.4 Hz, H-15), 1.27 (3H, s, H-18), 0.85 (3H, d, *J* = 6.4 Hz, H-16), 0.60 (3H, s, H-20), Naproxen moiety:δ 7.72 (1H, d, *J* = 8.4 Hz, H-6′), 7.70 (1H, d, *J* = 9.2 Hz, H-11′), 7.69 (1H, br s, H-4′), 7.44 (1H, br d, *J* = 8.4 Hz, H-12′), 7.17 (1H, dd, *J* = 9.2; 2.4 Hz, H-7′), 7.13 (1H, d, *J* = 2.4 Hz, H-9′), 3.93 (3H, s, OMe), 3.86 (1H, q, *J* = 7.2 Hz, H-2′), 1.60 (3H, d, *J* = 7.2 Hz, H-13′); ^13^C-NMR (CDCl_3_): δ 39.48 (C-1), 20.34 (C-2), 38.33 (C-3), 44.62 (C-4), 56.90 (C-5), 26.45 (C-6), 39.14 (C-7), 148.55 (C-8), 56.94 (C-9), 40.98 (C-10), 21.46 (C-11), 36.45 (C-12), 30.77 (C-13), 35.66 (C-14), 63.77 (C-15), 20.05 (C-16), 106.85 (C-17), 29.46 (C-18), 184.81 (C-19), 12.76 (C-20), Naproxen moiety: 175.21 (C-1′), 45.97 (C-2′), 136.25 (C-3′), 126.35 (C-4′), 129.33 (C-5′), 129.68 (C-6′), 119.39 (C-7′), 158.00 (C-8′), 105.97 (C-9′), 134.07 (C-10′), 127.51 (C-11′), 126.69 (C-12′), 18.91 (C-13′), 56.73 (OMe′); HR-MS (EI) *m*/*z* 533.3282 [M − H]^+^ (calcd. for C_34_H_45_O_5_, 533.3267).

*Imbricatol-15-yl naproxenate methyl ester* (**11**): Compound **10** (90 mg, 0.17 mmol), was methylated with a solution of CH_2_N_2_ in diethyl ether. The solvent was evaporated under reduced pressure and the residue was purified by silica gel column chromatography eluting with hexane/EtOAc (9:1) yielding 82 mg (89%) of **11**. Pale yellow resinous oil; Rf 0.63 (PE**/**EtOAc 9:1); ${\left[\alpha \right]}_{D}^{20}$ +9.2 (*c* 0.015, CHCl_3_); IR ν_max_ (film) 2944 (C–H, asymmetrical stretching, CH_3_ and CH_2_), 2868 (C–H, symmetrical stretching, CH_3_ and CH_2_), 1729 (C=O, ester), 1230, 1154 (C–O, ester) cm^−1^; ^1^H-NMR (CDCl_3_): δ 4.80, 4.41 (each, 1H, br s, H-17), 4.11 (2H, br t, *J* = 6.4 Hz, H-15), 3.64 (3H, s, OMe), 1.20 (3H, s, H-18), 0.85 (3H, d, *J* = 6.4 Hz, H-16), 0.48 (3H, s, H-20), Naproxen moiety: δ 7.71 (1H, d, *J* = 8.4 Hz, H-6′), 7.69 (1H, d, *J* = 9.2 Hz, H-11′), 7.68 (1H, br s, H-4′), 7.42 (1H, br d, *J* = 8.4 Hz, H-12′), 7.15 (1H, dd, *J* = 9.2; 2.4 Hz, H-7′), 7.12 (1H, d, *J* = 2.4 Hz, H-9′), 3.93 (3H, s, OMe), 3.86 (1H, q, *J* = 7.2 Hz, H-2′), 1.59 (3H, d, *J* = 7.2 Hz, H-13′); ^13^C-NMR (CDCl_3_): δ 39.53 (C-1), 20.39 (C-2), 38.67 (C-3), 44.70 (C-4), 56.73 (C-5), 26.66 (C-6), 39.17 (C-7), 148.67 (C-8), 56.93 (C-9), 40.72 (C-10), 21.45 (C-11), 36.44 (C-12), 30.78 (C-13), 35.63 (C-14), 63.75 (C-15), 20.03 (C-16), 106.72 (C-17), 29.24(C-18), 178.25 (C-19), 12.94 (C-20), 51.56 (OMe), Naproxen moiety: 175.16 (C-1′), 45.95 (C-2′), 136.25 (C-3′), 126.32 (C-4′), 129.32 (C-5′), 129.67 (C-6′), 119.36 (C-7′), 157.98 (C-8′), 105.95 (C-9′), 134.05 (C-10′), 127.48 (C-11′), 126.67 (C-12′), 18.91 (C-13′), 55.69 (OMe′); HR-MS (EI) *m*/*z* 549.3574 [M + H]^+^ (calcd. for C_35_H_49_O_5_, 549.3580).

*Oleanoyl ibuprofenate* (**12**): Compound **12** was synthesized as described for **6** from oleanolic acid and ibuprofen to afford, after purification by silica gel column chromatography eluting with hexane/EtOAc (8:2), 223 mg (62%) of **12**. Colorless resin; Rf 0.76 (PE**/**EtOAc 7:3); ${\left[\alpha \right]}_{D}^{20}$ +58.4 (*c* 0.29, CHCl_3_); IR ν_max_ (film) 3300–3400 (O–H, from carboxylic acid), 2947, 2925 (C–H, asymmetrical stretching, CH_3_ and CH_2_), 2874 (C–H, symmetrical stretching, CH_3_ and CH_2_), 1729 (C=O, ester), 1695 (C=O, carboxylic acid), 1169 (C–O, ester) cm^−1^; ^1^H-NMR (CDCl_3_): δ 5.28 (1H, br s, H-12), 4.45 (1H, dd, *J =* 11.6; 4.4 Hz, H-3α), 2.82 (1H, dd, *J =* 14.4; 3.1 Hz, H-18), 1.13 (3H, s), 0.91 (3H, s), 0.90 (3H, s), 0.88 (3H, s), 0.73 (3H, s), 0.71 (3H, s), 0.56 (3H, s), Ibuprofen moiety: δ 7.22 (2H, d, *J* = 8.0 Hz, H-4′ and H-8′), 7.10 (2H, d, *J* = 8.0 Hz, H-5′ and H-7′), 3.69 (1H, m, H-2′), 2.46 (2H, d, *J* = 7.2 Hz, H-10′), 1.50 (3H, d, *J* = 7.2 Hz, H-9′), 0.91 (6H, d, *J* = 6.0 Hz, H-12′ and H-13′); ^13^C-NMR (CDCl_3_): δ 38.06 (C-1), 27.69 (C-2), 80.88 (C-3), 37.89 (C-4), 55.28 (C-5), 18.10 (C-6), 32.59 (C-7), 39.27 (C-8), 47.51 (C-9), 36.90 (C-10), 23.40 (C-11), 122.28 (C-12), 143.78 (C-13), 41.64 (C-14), 29.71 (C-15), 23.53 (C-16), 46.72 (C-17), 41.30 (C-18), 45.85 (C-19), 30.69 (C-20), 33.87 (C-21), 32.39 (C-22), 27.95 (C-23), 16.83 (C-24), 15.30 (C-25), 17.86 (C-26), 25.91 (C-27), 184.30 (C-28), 33.11 (C-29), 23.64 (C-30), Ibuprofen moiety: 174.46 (C-1′), 45.58 (C-2′), 138.19 (C-3′), 129.18 (C-4′), 127.28 (C-5′), 140.33 (C-6′), 127.28 (C-7′), 129.18 (C-8′), 18.15 (C-9′), 45.04 (C-10′), 30.19 (C-11′), 22.35 (C-12′), 22.35 (C-13′); HR-MS (EI) *m*/*z* 662.5144 [M + NH_4_]^+^ (calcd. for C_43_H_68_NO_4_, 662.5148).

*Oleanoyl ibuprofenate methyl ester* (**13**): Compound **12** (100 mg, 0.15 mmol), was methylated with a solution of CH_2_N_2_ in diethyl ether. The solvent was evaporated under reduced pressure and the residue was purified by silica gel column chromatography eluting with hexane/EtOAc (9:1) yielding 91 mg (92%) of **13**. Colorless resin; Rf 0.79 (PE**-**EtOAc 9:1); ${\left[\alpha \right]}_{D}^{20}$ +54.7 (*c* 0.37, CHCl_3_); IR ν_max_ (film) 2947, 2931 (C–H, asymmetrical stretching, CH_3_ and CH_2_), 2871 (C–H, symmetrical stretching, CH_3_ and CH_2_), 1729 (C=O, ester), 1169 (C–O, ester) cm^−1^; ^1^H-NMR (CDCl_3_): δ 5.29 (1H, br s, H-12), 4.46 (1H, dd, *J =* 11.6; 4.4 Hz, H-3α), 3.63 (3H, s, OMe), 2.87 (1H, dd, *J =* 13.7; 3.5 Hz, H-18), 1.13 (3H, s), 0.92 (3H, s), 0.89 (3H, s), 0.87 (3H, s), 0.72 (3H, s), 0.71 (3H, s), 0.56 (3H, s), Ibuprofen moiety: δ 7.23 (2H, d, *J* = 8.2 Hz, H-4′ and H-8′), 7.09 (2H, d, *J* = 8.2 Hz, H-5′ and H-7′), 3.68 (1H, m, H-2′), 2.45 (2H, d, *J* = 7.2 Hz, H-10′), 1.51 (3H, d, *J* = 7.2 Hz, H-9′), 0.90 (6H, d, *J* = 6.0 Hz, H-12′ and H-13′); ^13^C-NMR (CDCl_3_): δ 38.16 (C-1), 27.31 (C-2), 81.27 (C-3), 37.28 (C-4), 55.63 (C-5), 18.27 (C-6), 32.77 (C-7), 39.65 (C-8), 47.89 (C-9), 36.64 (C-10), 23.45 (C-11), 122.67 (C-12), 144.18 (C-13), 41.67 (C-14), 28.37 (C-15), 23.58 (C-16), 46.20 (C-17), 40.12 (C-18), 45.97 (C-19), 30.12 (C-20), 33.52 (C-21), 31.09 (C-22), 27.96 (C-23), 16.87 (C-24), 15.70 (C-25), 17.22 (C-26), 25.67 (C-27), 178.71 (C-28), 32.96 (C-29), 23.79 (C-30), 51.93 (OMe), Ibuprofen moiety: 174.75 (C-1′), 45.63 (C-2′), 138.57 (C-3′), 129.59 (C-4′), 127.68 (C-5′), 140.75 (C-6′), 127.68 (C-7′), 129.59 (C-8′), 18.56 (C-9′), 45.07 (C-10′), 30.63 (C-11′), 22.70 (C-12′), 22.70 (C-13′); HR-MS (EI) *m*/*z* 676.5303 [M + NH_4_]^+^ (calcd. for C_44_H_70_NO_4_, 676.5305).

*Oleanoyl naproxenate* (**14**): Compound **14** was synthesized as described for **6** from oleanolic acid and naproxen to afford, after purification by silica gel column chromatography eluting with hexane/EtOAc (85:25), 215 mg (61%) of **14**. Colorless resin; Rf 0.56 (PE**/**EtOAc 6:4); ${\left[\alpha \right]}_{D}^{20}$ +60.6 (*c* 0.180, CHCl_3_); IR ν_max_ (film) 3300–3400 (O–H, from carboxylic acid), 2938 (C–H, asymmetrical stretching, CH_3_ and CH_2_), 2849 (C–H, symmetrical stretching, CH_3_ and CH_2_), 1732 (C=O, ester), 1689 (C=O, carboxylic acid), 1269, 1178 (C–O, ester) cm^−1^; ^1^H-NMR (CDCl_3_): δ 5.25 (1H, br s, H-12), 4.46 (1H, dd, *J =* 11.6; 4.4 Hz, H-3α), 2.82 (1H, dd, *J =* 13.6; 3.5 Hz, H-18), 1.11 (3H, s), 0.92 (3H, s), 0.89 (6H, s), 0.71 (6H, s), 0.58 (3H, s), Naproxen moiety: δ 7.68 (2H, br d, *J* = 8.6 Hz, H-6′ and H-11′), 7.67 (1H, br s, H-4′), 7.41 (1H, br d, *J* = 8.8 Hz, H-12′), 7.12 (1H, dd, *J* = 9.0; 2.0 Hz, H-7′), 7.10 (1H, d, *J* = 2.4 Hz, H-9′), 3.90 (3H, s, OMe), 3.85 (1H, q, *J* = 7.2 Hz, H-2′), 1.57 (3H, d, *J* = 7.2 Hz, H-13′); ^13^C-NMR (CDCl_3_): δ 38.02 (C-1), 27.64 (C-2), 81.11 (C-3), 37.87 (C-4), 55.27 (C-5), 18.03 (C-6), 32.50 (C-7), 39.26 (C-8), 47.51 (C-9), 36.94 (C-10), 23.38 (C-11), 122.52 (C-12), 143.59 (C-13), 41.55 (C-14), 29.72 (C-15), 23.53 (C-16), 46.54 (C-17), 40.92 (C-18), 45.83 (C-19), 30.66 (C-20), 33.80 (C-21), 32.44 (C-22), 27.72 (C-23), 16.56 (C-24), 15.32 (C-25), 17.10 (C-26), 25.91 (C-27), 184.37 (C-28), 33.07 (C-29), 23.58 (C-30), Naproxen moiety: 174.29 (C-1′), 46.01 (C-2′), 136.04 (C-3′), 126.04 (C-4′), 128.94 (C-5′), 129.26 (C-6′), 118.88 (C-7′), 157.56 (C-8′), 105.61 (C-9′), 133.65 (C-10′), 126.99 (C-11′), 126.40 (C-12′), 18.23 (C-13′), 55.31 (OMe′); HR-MS (EI) *m*/*z* 686.4766 [M + NH_4_]^+^ (calcd. for C_44_H_64_NO_5_, 686.4784).

*Oleanoyl naproxenate methyl ester* (**15**): Compound **14** (100 mg, 0.15 mmol), was methylated with a solution of CH_2_N_2_ in diethyl ether. The solvent was evaporated under reduced pressure and the residue was purified by silica gel column chromatography eluting with hexane/EtOAc (95:5) yielding 84 mg (85%) of **15**. Colorless resin; Rf 0.23 (PE**-**EtOAc 9:1); ${\left[\alpha \right]}_{D}^{20}$ +66.6 (*c* 0.37, CHCl_3_); IR ν_max_ (film) 2950 (C–H, asymmetrical stretching, CH_3_ and CH_2_), 2846 (C–H, symmetrical stretching, CH_3_ and CH_2_), 1722 (C=O, ester), 1260, 1184 (C–O, ester) cm^−1^; ^1^H-NMR (CDCl_3_): δ 5.26 (1H, br s, H-12), 4.46 (1H, dd, *J =* 11.6; 4.4 Hz, H-3α), 3.61 (3H, s, OMe), 2.81 (1H, dd, *J =* 13.7; 3.2 Hz, H-18), 1.10 (3H, s), 0.92 (3H, s), 0.89 (6H, s), 0.70 (3H, s), 0.69 (3H, s), 0.57 (3H, s), Naproxen moiety: δ 7.69 (1H, d, *J* = 8.4 Hz, H-6′), 7.68 (1H, d, *J* = 9.2 Hz, H-11′), 7.67 (1H, br s, H-4′), 7.40 (1H, br d, *J* = 8.4 Hz, H-12′), 7.13 (1H, dd, *J* = 9.2; 2.4 Hz, H-7′), 7.11 (1H, d, *J* = 2.4 Hz, H-9′), 3.90 (3H, s, OMe), 3.85 (1H, q, *J* = 7.2 Hz, H-2′), 1.57 (3H, d, *J* = 7.2 Hz, H-13′); ^13^C-NMR (CDCl_3_): δ 38.05 (C-1), 27.67 (C-2), 81.09 (C-3), 37.87 (C-4), 55.27 (C-5), 18.08 (C-6), 32.56 (C-7), 39.26 (C-8), 47.50 (C-9), 36.89 (C-10), 23.39 (C-11), 122.26 (C-12), 143.79 (C-13), 41.63 (C-14), 29.71 (C-15), 23.53 (C-16), 46.72 (C-17), 41.30 (C-18), 45.84 (C-19), 30.68 (C-20), 33.85 (C-21), 32.38 (C-22), 27.70 (C-23), 16.57 (C-24), 15.29 (C-25), 16.82 (C-26), 25.89 (C-27), 178.30 (C-28), 33.10 (C-29), 23.63 (C-30), 51.51 (OMe), Naproxen moiety: 174.26 (C-1′), 45.99 (C-2′), 136.04 (C-3′), 126.00 (C-4′), 128.93 (C-5′), 129.25 (C-6′), 118.86 (C-7′), 157.54 (C-8′), 105.59 (C-9′), 133.62 (C-10′), 126.96 (C-11′), 126.40 (C-12′), 18.16 (C-13′), 55.31 (OMe′); HR-MS (EI) *m*/*z* 683.7180 [M + H]^+^ (calc. for C_45_H_63_O_5_, 683.4676).

### 3.3. Topical Anti-Inflammatory Effect

The inflammatory agent arachidonic acid (AA) and phorbol 12-myristate 13-acetate (TPA) were used to assess the anti-inflammatory effect/action mechanism of the starting compounds, new synthetic products and reference drugs. All animal experiments were performed according to the ethical guidelines suggested by the “International Norms for the Biomedical Investigation with Animals”, elaborated by the Council of International Organizations (1990) and the bio-ethics norms of the Commission of the Chilean Public Health Institute and Facultad de Ciencias Químicas y Farmacéuticas, Universidad de Chile (certification number CBE2014-4). Adult male CF-1 mice (20–25 g), obtained from a stock maintained at the Chilean Public Health Institute, were used to assess the anti-inflammatory effect. All animals were housed in a climate- and light-controlled room with a 12 h light-dark cycle, fasted overnight before the day of the assays, with free access to water. The anti-inflammatory activity of each compound was evaluated in groups of 8 treated and 16 control mice. Due to ethical considerations, the number of animals was kept to a minimum and single dose experiments were carried out.

The pro-inflammatory agents TPA and AA, the reference drugs indomethacin and nimesulide were from Sigma. Mice were pre-treated with compounds **1**–**15** or reference drugs at the same equimolar dose (3.2 µmol/mouse for nimesulide and 1.4 µmol/mouse for indomethacin). After 5 min, inflammation was induced by the application of 20 µL TPA or AA (5 mg/mL) in acetone. The solvent does not interfere with the assay. Control subjects only received TPA or AA. Both, the sample and the TPA or AA, were applied to the inner (10 µL) and outer (10 µL) surfaces of the right ear. The left ear only received acetone. Mice were sacrificed by cervical dislocation (after 6 h of TPA and 1 h AA), and a 6 mm diameter section of the right and left ears were cut and weighed. The weight differences between both ear sections correspond to the edema value [31]. The percent topical anti-inflammatory activity (TA) was evaluated according to the following equation:

(1) $$\%TA=\left[\;\frac{Wc-Ws}{Wc}\right]\times \;100$$

where *Wc* and *Ws* are the different median values of the weights of the right and the left ear sections of the control and the treated animals, respectively [32].

### 3.4. Cytotoxicity Assay

The human cell lines, MRC-5 normal lung fibroblasts (CCL-171), AGS gastric epithelial adenocarcinoma cells (CRL-1739) and HepG2 hepatocellular carcinoma cells (HB-8065) were obtained from the American Type Culture Collection (ATCC, Manasas, VA, USA). The cells were grown as monolayers in the following media: MRC-5 and HepG2 in MEM and AGS in Ham F-12. The MEM medium contained 2 mM l-glutamine, 1 mM sodium pyruvate and 1.5 g/L sodium bicarbonate. Ham F-12 was supplemented with 2 mM l-glutamine and 1.5 g/L sodium bicarbonate. Both media were supplemented with 10% heat inactivated fetal bovine serum, 100 IU/mL penicillin and 100 µg/mL streptomycin in a humidified incubator with 5% CO_2_ in air at 37 °C. For the experiments, cells were plated at a density of 25,000 cells/mL in 96-well plates. Confluent cultures of the different cell lines were treated with medium containing the compounds at concentrations ranging from 0 up to 1000 µM. Compounds were first dissolved in DMSO and then in medium. The final concentration of DMSO in the test medium and controls was 1%. Final concentrations of DMSO did not interfere with cell growth. Cells were exposed to test medium for 24 h, with or without the compound (control). In the experiments, the 100% viability controls were cells treated with medium only. The cell viability for each compound was calculated comparing the cell viability of the untreated controls (100%) with the cell viability of the different concentrations of each compound. Each concentration was tested in quadruplicate together with the control and repeated three times in separate experiments. Cell viability was determined at the end of the incubation by means of the MTT reduction assay [33]. Results were converted to percentage of controls, and the IC_50_ values were graphically obtained from the dose-response curves. Culture media, antibiotics and fetal bovine serum were obtained from Invitrogen Corp. (Waltham, MA, USA). Other reagents were purchased from Sigma Chemical Co.

### 3.5. Statistical Analysis

For the animal experiments all values are presented as median ± SEM. Drug-induced changes were statistically estimated using the Kruskall-Wallis test for comparison between groups and testing, and the Mann-Whitney test was used in the independent data for individual comparisons. The effects were considered significant if *p* ≤ 0.05. For cytotoxicity experiments, each concentration was tested in quadruplicate together with the control and repeated three times in different experiments. Results are expressed as IC_50_ values (µM) ± S.D.

## 4. Conclusions

Starting from three naturally occurring anti-inflammatory terpenes and two synthetic drugs, 10 derivatives were synthesized, combining the terpenyl and anti-inflammatory moieties into a single new chemical entity. The anti-inflammatory effect of the starting compounds and new products was compared at the same equimolar dose using two different assays for topic anti-inflammatory effect. In the AA-induced inflammation, the new ferruginyl naproxenate (**7**) and oleanoyl ibuprofenate (**12**) showed a remarkable increase in activity, compared with the single terpene and synthetic anti-inflammatory drugs. Both compounds **7** and **12** showed a strong decrease in cytotoxicity compared with that of the single terpenes in the three cell lines. The results suggest that new products containing a terpenyl and a synthetic moiety may present better properties than the single chemical entities in selected biological assays. Additional studies are needed to disclose the therapeutical potential of the new anti-inflammatory agents as well as to assess their mechanisms of action.
